# Economic Impact of Adverse Drug Events – A Retrospective Population-Based Cohort Study of 4970 Adults

**DOI:** 10.1371/journal.pone.0092061

**Published:** 2014-03-17

**Authors:** Hanna Gyllensten, Katja M. Hakkarainen, Staffan Hägg, Anders Carlsten, Max Petzold, Clas Rehnberg, Anna K. Jönsson

**Affiliations:** 1 Nordic School of Public Health NHV, Gothenburg, Sweden; 2 Section of Social Medicine, Department of Public Health and Community Medicine, Sahlgrenska Academy, University of Gothenburg, Gothenburg, Sweden; 3 Department of Drug Research/Clinical Pharmacology, Faculty of Health Sciences, Linköping University, Linköping, Sweden; 4 Futurum – Academy for Health and Care, Jönköping County Council, Jönköping, Sweden; 5 Medical Products Agency, Uppsala, Sweden; 6 Akademistatistik – Centre for Applied Biostatistics, Occupational and Environmental Medicine, Sahlgrenska Academy, University of Gothenburg, Gothenburg, Sweden; 7 Department of Learning, Informatics, Management and Ethics – LIME, Karolinska Institutet, Stockholm, Sweden; 8 Department of Clinical Pharmacology, County Council of Östergötland, Linköping, Sweden; Dana-Farber Cancer Institute, United States of America

## Abstract

**Background:**

The aim was to estimate the direct costs caused by ADEs, including costs for dispensed drugs, primary care, other outpatient care, and inpatient care, and to relate the direct costs caused by ADEs to the societal COI (direct and indirect costs), for patients with ADEs and for the entire study population.

**Methods:**

We conducted a population-based observational retrospective cohort study of ADEs identified from medical records. From a random sample of 5025 adults in a Swedish county council, 4970 were included in the analyses. During a three-month study period in 2008, direct and indirect costs were estimated from resource use identified in the medical records and from register data on costs for resource use.

**Results:**

Among 596 patients with ADEs, the average direct costs per patient caused by ADEs were USD 444.9 [95% CI: 264.4 to 625.3], corresponding to USD 21 million per 100 000 adult inhabitants per year. Inpatient care accounted for 53.9% of all direct costs caused by ADEs. For patients with ADEs, the average societal cost of illness was USD 6235.0 [5442.8 to 7027.2], of which direct costs were USD 2830.1 [2260.7 to 3399.4] (45%), and indirect costs USD 3404.9 [2899.3 to 3910.4] (55%). The societal cost of illness was higher for patients with ADEs compared to other patients. ADEs caused 9.5% of all direct healthcare costs in the study population.

**Conclusions:**

Healthcare costs for patients with ADEs are substantial across different settings; in primary care, other outpatient care and inpatient care. Hence the economic impact of ADEs will be underestimated in studies focusing on inpatient ADEs alone. Moreover, the high proportion of indirect costs in the societal COI for patients with ADEs suggests that the observed costs caused by ADEs would be even higher if including indirect costs. Additional studies are needed to identify interventions to prevent and manage ADEs.

## Introduction

Adverse drug events (ADEs), ”injuries resulting from medical intervention related to a drug”,[1] cause significant harm to patients and increased resource use. It has been estimated that 5–6% of all hospitalisations are drug-related,[2], [3] and the additional hospitalisation costs of patients experiencing ADEs have been estimated to USD 2284–5640 per patient (2000 values).[4] However, indirect costs, costs in the general population, and costs in outpatient settings, caused by ADEs, have not been included. Hence the costs caused by ADEs are largely unknown.[4], [5]

In a Swedish survey study of the adult general public, respondents reporting ADEs during one month had a 150% higher overall cost of illness (COI) compared to respondents without ADEs, and considerable direct costs caused by ADEs.[6] However, these costs based on survey data may be underestimated, as the respondents probably had fewer healthcare encounters than non-respondents.

Thus we conducted a study to examine ADEs identified from medical records in a random sample of the adult general population. The aim of this study was to estimate the direct costs caused by ADEs, including costs for dispensed drugs, primary care, other outpatient care, and inpatient care, and to relate the direct costs caused by ADEs to the societal COI (direct and indirect costs), for patients with ADEs and for the entire study population.

## Methods

### Ethics statement

The study received ethical approval from the Regional Ethical Review Board in Gothenburg (approval reference number: 644–08). Informed consent was not obtained since the retrospective study did not affect the healthcare of the included individuals. Although personal identity numbers were used to link data from medical records and registers, data were analysed anonymously. Statistics Sweden replaced the personal identity numbers by a random serial number after the final data linkage.

### Study design

We conducted a population-based observational retrospective cohort study to examine ADEs identified from medical records from primary care, other outpatient care and inpatient care. Identified ADEs included: adverse drug reactions, drug abuse, drug dependence, drug intoxications from overdose, sub-therapeutic effect of drug therapy, and morbidity due to drug-related untreated indication.[7] During a three-month study period in 2008, information on direct and indirect costs was obtained from the medical records and register data.

### Participants and data collection

In depth description of the sample size calculation, identification, exclusion and evaluation process is reported elsewhere.[7] In brief, a random sample of 5025 individuals 18 years or older on 31 December 2007 were identified by Statistics Sweden from the Östergötland County adult population, a county with 335 780 adult inhabitants in 2008.[8] To manage seasonal variation, the study population was randomly divided into four groups and each group was allotted a three-month study period during 2008.

**Figure 1:**
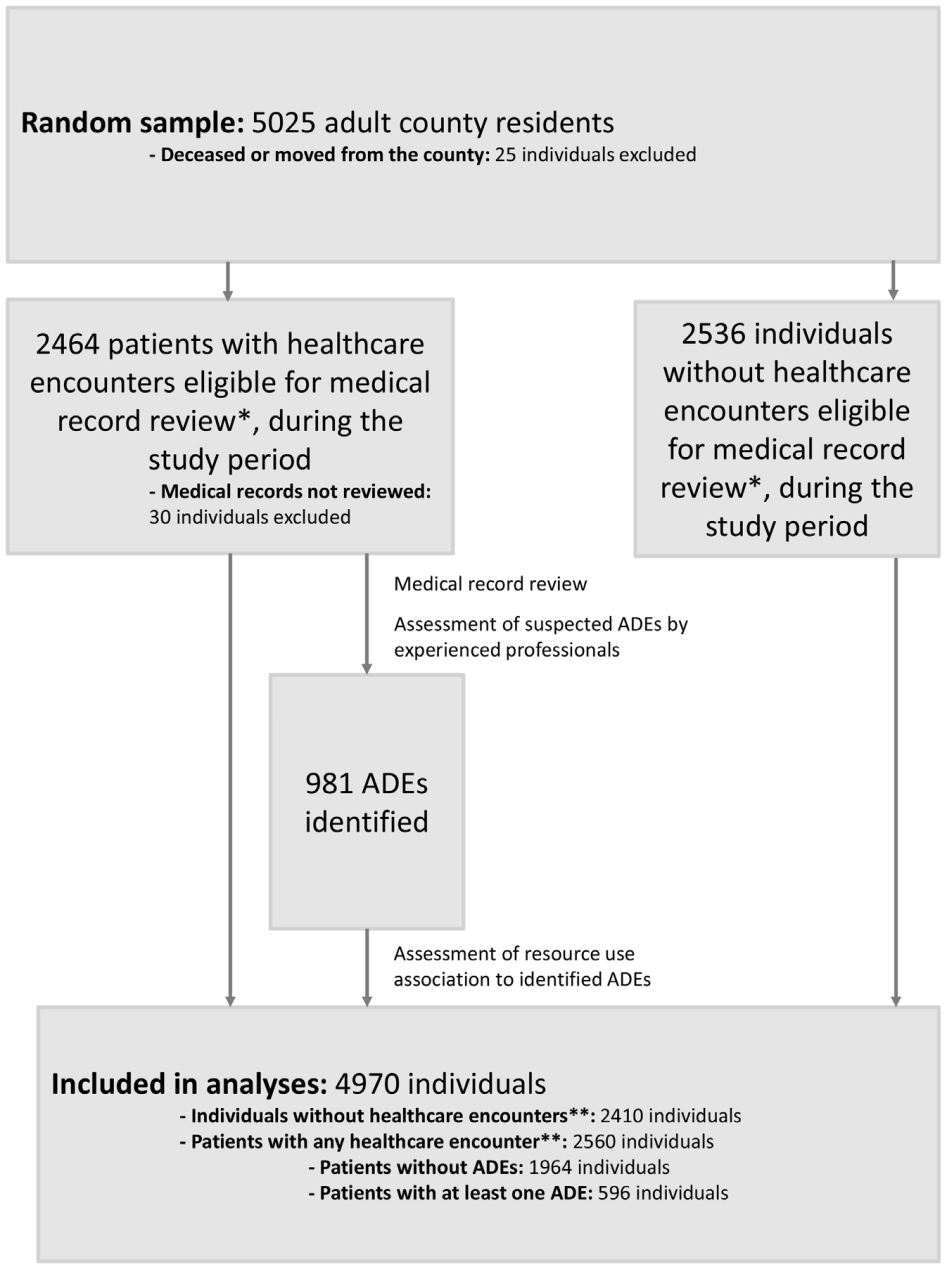
Data collection to identify adverse drug events (ADE) from medical records. * Hospitalisations or outpatient physician or nurse encounters. ** In addition to hospitalisations and outpatient physician or nurse encounters, any healthcare encounter includes also other outpatient encounters (that were not eligible for the medical record review), e.g. physiotherapist and nutritionist encounters.

The healthcare encounters of individuals still living in the county during the allotted study period (n  =  5000) were identified from the Care Data Warehouse, the regional patient register in Östergötland County that includes all public and most private healthcare encounters. Data on prescribed and dispensed drugs were obtained from medical records and the Swedish Prescribed Drug Register of the National Board of Health and Welfare. A pharmacist manually reviewed the medical records of individuals with hospitalisations or outpatient physician or nurse encounters during the study period (n  =  2464), including medical records before and after the study period. From the medical records, using triggers and a list of dispensed drugs from four months before to one month after the study period, the pharmacist collected data on suspected ADEs in a standardised data collection sheet. The suspected ADEs were evaluated independently by two experienced professionals, one physician and one pharmacist, according to published criteria for causality,[9] preventability,[10] and contribution to hospitalisations.[10] The individual evaluations were discussed to reach consensus. ADEs judged definite, probable or possible[9] were categorised as preventable [10] (definitely or possibly) or not, and categorised as adverse drug reactions, drug abuse, drug dependence, drug intoxications from overdose, sub-therapeutic effect of drug therapy, and morbidity due to drug-related untreated indication.[7] After the medical record review, 4970 individuals were included in the final analyses (figure 1).

Using personal identity numbers, data from the medical records, the Care Data Warehouse, and the Swedish Prescribed Drug Register were linked to additional register data: demographic and socioeconomic variables (age, sex, country of birth, education, marital status, main occupation, and disposable income) from Statistics Sweden, sick-leave and disability pension from the Social Insurance Agency, and administrative data from the Cost Per Patient Register from Östergötland County Council. Main occupation was derived from register data, assuming that the sources and distribution of income reflected the individuals' occupations (e.g. if the main income was from student grants the occupation was interpreted as student).

### Direct costs caused by ADEs

For patients with ADEs, each healthcare encounter during the study period was evaluated for costs caused by prevalent ADEs. The association between the ADEs and the healthcare encounter was evaluated as dominant (i.e. the main reason for the encounter), partly contributing (i.e. played a substantial role for the encounter), less important (i.e. played a minor or uncertain role for the encounter), or not contributing (i.e. other symptoms/circumstances were the main reason for the encounter). The categories were based on Hallas' criteria for drug-related hospitalisations.[10] For encounters with at least “less important” association, the costs of diagnosing, treating, and monitoring ADEs included healthcare use and costs of dispensed drugs assigned from the Cost Per Patient Register or the Swedish Prescribed Drug Register. The direct costs caused by ADEs consisted of the full costs of healthcare encounters dominantly caused by ADEs, and additionally the costs of diagnosing, treating or monitoring ADEs when the entire encounter was not dominantly caused by ADEs. If costs were not identified in the register, the resource use was reported descriptively.

### Direct and indirect costs in the societal COI

COI studies measure the economic impact of disease and can be used to inform decision makers of the financial relevance in relation to other health issues.[11] COI can be estimated from the societal perspective, which includes both direct and indirect costs regardless of who holds the expense.[12] Direct costs are the costs of used resources (e.g. in healthcare), and indirect costs are costs due to loss of productivity.[11]

For the societal COI, direct and indirect costs were estimated for all study individuals. The direct costs included costs of healthcare services from the Cost Per Patient Register and dispensed drugs (both individuals' out-of-pocket costs and reimbursements) from the Swedish Prescribed Drug Register. Data for indirect costs were extracted from the Swedish Social Insurance Agency and included individuals' lost productivity due to sick-leave or disability pension, estimated by the human capital approach,[11] based on the age-specific national wages statistics and compulsory social security contributions.[13], [14]

### Analyses

#### Description of the study population

Differences in the socio-demographic characteristics of patients with ADEs compared to patients without ADEs, and all patients compared to individuals without any healthcare encounters, were tested for statistical significance (at p<0.05) using Fisher's exact test, or chi square test when exceeding the memory limit of Fisher's exact test. All cost analyses were prevalence-based, i.e. included costs during the study period for each patient, and were translated to United States dollar (USD) using the 2008 exchange rate (USD 1  =  SEK 6.5808[15]). All cost estimates were described with 95% confidence interval.

#### Analyses of direct costs caused by ADEs

For patients with ADEs, the average total direct costs caused by ADEs per person with ADEs were reported descriptively. The average direct costs for encounters dominantly caused by ADEs and for encounters with interventions for diagnosing, treating, or monitoring ADEs were also described, to enable comparisons to other studies. Interventions for diagnosing, treating or monitoring ADEs without an assigned cost in the register were presented descriptively. The distribution of healthcare encounters with direct costs caused by ADEs was also reported by care level: in primary care, other outpatient care (e.g. specialist physician visits and outpatient clinics in hospital) and inpatient care. The overall direct costs for ADEs per 100 000 adult inhabitants per year were calculated from the direct costs resulting from all ADEs during the three months, as following: [(direct costs per person with ADEs * total number of persons with ADEs)/all study individuals] * 4 * 100 000. The multiplication by four was for extrapolating three-month costs to annual costs.

#### Analyses of directs costs caused by ADEs in relation to societal COI

For patients with ADEs, direct and indirect costs were described and tested for statistical significance (p<0.05) compared to patients without ADEs, using two-tailed t-tests with unequal variances. Since the cost data were skewed, Wilcoxon rank-sum test was also used, but with minimal changes to the statistical significances. For patients with ADEs, the direct costs caused by ADEs and the societal COI, were presented by patient and ADE characteristics. Differences by age categories were tested for statistical significance (p<0.05) using one-way Anova, and by sex using two-tailed t-tests with unequal variances. The average direct healthcare costs per person were also illustrated by care level (dispensed drugs, primary care, other outpatient care, and inpatient care) for the study population, all patients with healthcare encounters, total costs for patients with ADEs, and for costs caused by ADEs among patients with ADEs.

#### Sensitivity analysis

A sensitivity analysis of the direct costs was conducted based on Diagnosis Related Groups[16] (DRG; a system for classifying patients on resource use, based on diagnoses, procedures performed, age, sex and status at discharge). The DRG weight of each encounter was identified from the Care Data Warehouse. Differences in DRG weight per encounter, between patients with and without ADEs, were tested for statistical significance (p<0.05) using a two-tailed t-tests with unequal variances.

Statistical analyses were made using the STATA/IC statistical software, version 11.2.

## Results

ADEs were identified among 596 (12.0%) of the 4970 individuals in the study population. The characteristics of the study population are presented in table 1. The identified ADEs are further described elsewhere.[7]

**Table 1:** Characteristics of the study population, compared for patients with/without adverse drug events (ADEs), and for all patients compared to individuals without healthcare encounters.

	Patients* with ADEs	Patients* without ADEs	Individuals without any healthcare encounters
	n (%)	n (%)	n (%)
**Age**	n = 596	n = 1964, p<0.001	n = 2410, p<0.001
18–34 years	73 (12.2)	464 (23.6)	843 (35.0)
35–64 years	267 (44.8)	913 (46.5)	1257 (52.1)
≥65 years	256 (43.0)	587 (29.9)	310 (12.9)
**Sex**	n = 596	n = 1964, p = 0.727	n = 2410, p<0.001
Men	238 (39.9)	800 (40.7)	1389 (57.6)
Women	358 (60.1)	1164 (59.3)	1021 (42.4)
**Countryofbirth**	n = 596	n = 1964, p = 0.341	n = 2409, p = 0.021
Sweden	532 (89.3)	1779 (90.6)	2126 (88.3)
Outside Sweden	64 (10.7)	185 (9.4)	283 (11.7)
**Maritalstatus**	n = 596	n = 1964, p<0.001	n = 2410, p<0.001
Single	137 (23.0)	650 (33.1)	1127 (46.7)
Married or registered partnership	295 (49.5)	930 (47.4)	978 (40.6)
Divorced	80 (13.4)	213 (10.8)	233 (9.7)
Widowed	84 (14.1)	171 (8.7)	72 (3.0)
**Education**	n = 571	n = 1928, p<0.001	n = 2368, p<0.001
Mandatory education	242 (42.4)	550 (28.5)	472 (19.9)
Intermediate education	234 (41.0)	911 (47.3)	1262 (53.3)
High education	95 (16.6)	467 (24.2)	634 (26.8)
**Mainoccupation**	n = 577	n = 1945, p<0.001†	n = 2392, p<0.001†
Employee	174 (30.2)	953 (49.0)	1589 (66.4)
Company owner	8 (1.4)	48 (2.5)	96 (4.0)
Student	7 (1.2)	72 (3.7)	141 (5.9)
Retired	240 (41.6)	572 (29.4)	305 (12.8)
On long-term sickness absence or disability pensioner	115 (19.9)	183 (9.4)	82 (3.4)
Other	33 (5.7)	117 (6.0)	179 (7.5)
**Disposableincomein 2008, quartiles (USD)**	n = 576	n = 1945, p = 0.002	n = 2392, p<0.001
<16031	162 (28.1)	486 (25.0)	584 (24.4)
16031–21806	174 (30.2)	528 (27.1)	526 (22.0)
21806–31394	146 (25.3)	466 (24.0)	613 (25.6)
>31394	94 (16.3)	465 (23.9)	669 (28.0)

* Individuals with any healthcare encounters.

† Tested for statistical significance by chi square test, since the data exceeded the memory limit for Fisher's exact test.

### Direct costs caused by ADEs

**Figure 2:**
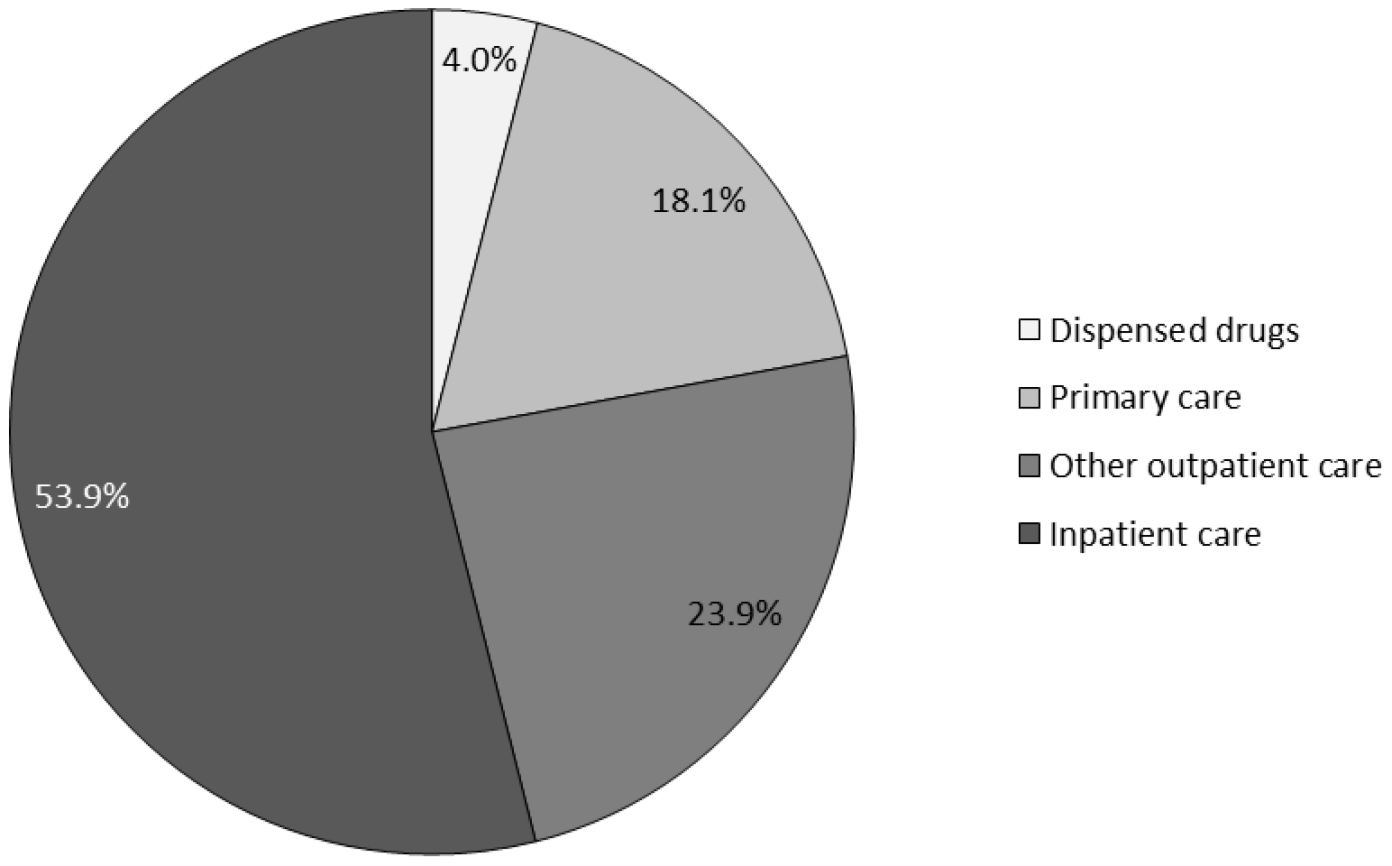
Distribution of direct costs caused by adverse drug events (ADE), by care level.

ADEs were assessed as a dominant cause of 991 healthcare encounters (table 2), causing 15.9% of all 189 hospitalisations and 18.0% of all 206 somatic emergency department visits in the study population during the three months. Interventions for diagnosing, treating or monitoring ADEs were initiated in 2016 encounters (table 3), of which 1025 (50.8%) were not dominantly caused by ADEs. Among the 596 patients with at least one ADE, the average direct costs per patient caused by ADEs were (mean [95% confidence interval]) USD 444.9 [264.4 to 625.3], including the full cost of the 991 healthcare encounters dominantly caused by ADEs and the costs of interventions for diagnosing, treating or monitoring ADEs in the 1025 additional encounters. This corresponds to overall direct costs of USD 21 million per 100 000 adult inhabitants per year. The costs of inpatient care accounted for 53.9% of all direct costs caused by ADEs (figure 2).

**Table 2:** Healthcare encounters dominantly caused (991 encounters) by adverse drug events (ADEs) and the resulting direct costs per encounter.

Encounters	Encounters dominantly caused by ADEs, by care level			Average direct costs caused by ADEs, per encounter*	Resource use for ADEs in the encounters
	Primary care	Other out-patient care	Inpatient care	USD (95% CI)	Cause (No of encounters)
Telephone contacts	128	58	0	26.9 (16.9 to 36.9)	Diagnosis (100)
					Treatment (77)
					Monitoring (70)
Nurse visits	164	44	0	49.4 (41.0 to 57.8)	Diagnosis (67)
					Treatment (67)
					Monitoring (117)
Physicians visits	252	0	0	117.3 (102.1 to 132.5)	Diagnosis (141)
					Treatment (149)
					Monitoring (96)
Specialist physician visits	0	157	0	308.9 (264.5 to 353.3)	Diagnosis (88)
					Treatment (87)
					Monitoring (60)
Home healthcare	40	21	0	266.4 (171.0 to 361.8)	Diagnosis (15)
					Treatment (22)
					Monitoring (40)
Other outpatient visits	60	37	0	78.4 (55.1 to 101.7)	Diagnosis (13)
					Treatment (60)
					Monitoring (31)
Hospitalisations	0	0	30	6209.0 (1868.3 to 10 549.7)	Diagnosis (20)
					Treatment (19)
					Monitoring (12)
**Total**	**644**	**317**	**30**	–	
% of healthcare encounters dominantly caused by ADEs	65.0	32.0	3.0	–	

* The cost of the entire encounter included in the direct costs caused by ADEs. Excluding encounters in private healthcare when not included in the Cost Per Patient Register.

**Table 3:** Healthcare encounters (2016 encounters) with interventions for diagnosing, treating and monitoring adverse drug events (ADEs), and the resulting direct costs per encounter.

Interventions	Encounters for which ADEs were dominantly, partly or less contributing, by care level			Average direct costs caused by ADEs, per encounter*
Level of association (No of encounters)	Primary care	Other out-patient care	Inpatient care	USD mean (95% CI)
**Interventionsfor** ***diagnosing*** ** ADEs**				
Dominant (444); partly (184); less (275)				
Laboratory tests	44	22	22	78.4 (56.6 to 100.3)
Other examinations	2	4	5	311.0 (90.2 to 531.8)
**Interventionsfor** ***treating*** ** ADEs**				
Dominant (487); partly (200); less (141)				
Dispensed drugs	146	70	12	35.7 (27.0 to 44.4)‡
Prescribing of drugs associated with abuse or dependence	20†	–	–	119.1 (22.1 to 216.1)
Other treatments	82	0	0	85.4 (78.0 to 92.8)
**Interventionsfor** ***monitoring*** ** ADEs**				
Dominant (426); partly (303); less (133)				
Laboratory tests	16	7	8	67.9 (38.3 to 97.5)
Other examinations	3	4	2	303.6 (−58.5 to 665.7)

* The cost of the intervention included in the direct costs caused by ADEs. Excluding encounters, and costs for interventions not identified in registers.

† Prescribing of drugs associated with abuse or dependence were not analysed according to care level, since prescriber data was not available in the Swedish Prescribed Drug Register.

‡ In addition, two individuals were prescribed medicines (total cost: USD 140.6) for treating ADEs during healthcare encounters that were not identified in the Cost Per Patient Register.

### Direct and indirect costs in the societal COI

Among the study population, 2560 patients accounted for 13 914 healthcare encounters during the study period. Of all encounters, 1845 (13.3%) were not assigned a cost. The average societal COI for patients with ADEs were USD 6235.0 [5442.8 to 7027.2] (table 4), of which direct costs were USD 2830.1 [2260.7 to 3399.4] (45%), and indirect costs USD 3404.9 [2899.3 to 3910.4] (55%). The total societal COI, and its direct and indirect costs, was higher per patient with ADEs than per patient without ADEs during the three-month study period.

**Table 4:** The average societal cost of illness (COI) per patient with and without adverse drug events (ADEs) (n = 2560).

Components of societal COI	Societal COI per patient with ADEs	Societal COI per patient without ADEs	Per patient cost difference in societal COI between patients with and without ADEs
	n = 596, USD mean (95% CI)	n = 1964, USD mean (95% CI)	USD mean (95% CI), p-value
Dispensed drugs *	306.6 (266.1 to 347.0)	176.4 (151.0 to 201.8)	130.2 (82.5 to 177.9), p<0.001
Primary healthcare use	457.6 (396.0 to 519.1)	188.5 (163.7 to 214.1)	268.7 (202.2 to 335.1), p<0.001
Other outpatient healthcare use	829.6 (647.2 to 1011.9)	254.3 (214.1 to 294.5)	575.2 (388.6 to 761.9), p<0.001
Inpatient healthcare use	1240.0 (782.9 to 1697.1)	157.8 (102.5 to 213.0)	1082.2 (621.8 to 1542.7), p<0.001
**Totaldirectcost**	**2830.1 (2260.7 to 3399.4)**	**754.5 (670.8 to 838.2)**	**2075.6 (1500.1 to 2651.0), p<0.001**
Productivity loss from sick-leave	794.0 (549.1 to 1038.9)	394.2 (299.5 to 488.9)	399.8 (137.3 to 662.3), p = 0.003
Productivity loss from disability pension	2610.9 (2140.8 to 3081.0)	1291.4 (1106.5 to 1476.2)	1319.5 (814.5 to 1824.5), p<0.001
**Totalindirectcost**	**3404.9 (2899.3 to 3910.4)**	**1685.6 (1480.6 to 1890.5)**	**1719.3 (1174.0 to 2264.6), p<0.001**
**Totalsocietal COI**	**6235.0 (5442.8 to 7027.2)**	**2440.1 (2210.3 to 2669.9)**	**3794.9 (2970.2 to 4619.6), p<0.001**

* Average cost of dispensed drugs for 589 patients with ADEs and 1710 patients without ADEs, the remaining 7 patients with ADEs and 254 patients without ADEs did not use dispensed drugs during the study period.

### Direct costs caused by ADEs in relation to the societal COI

**Figure 3:**
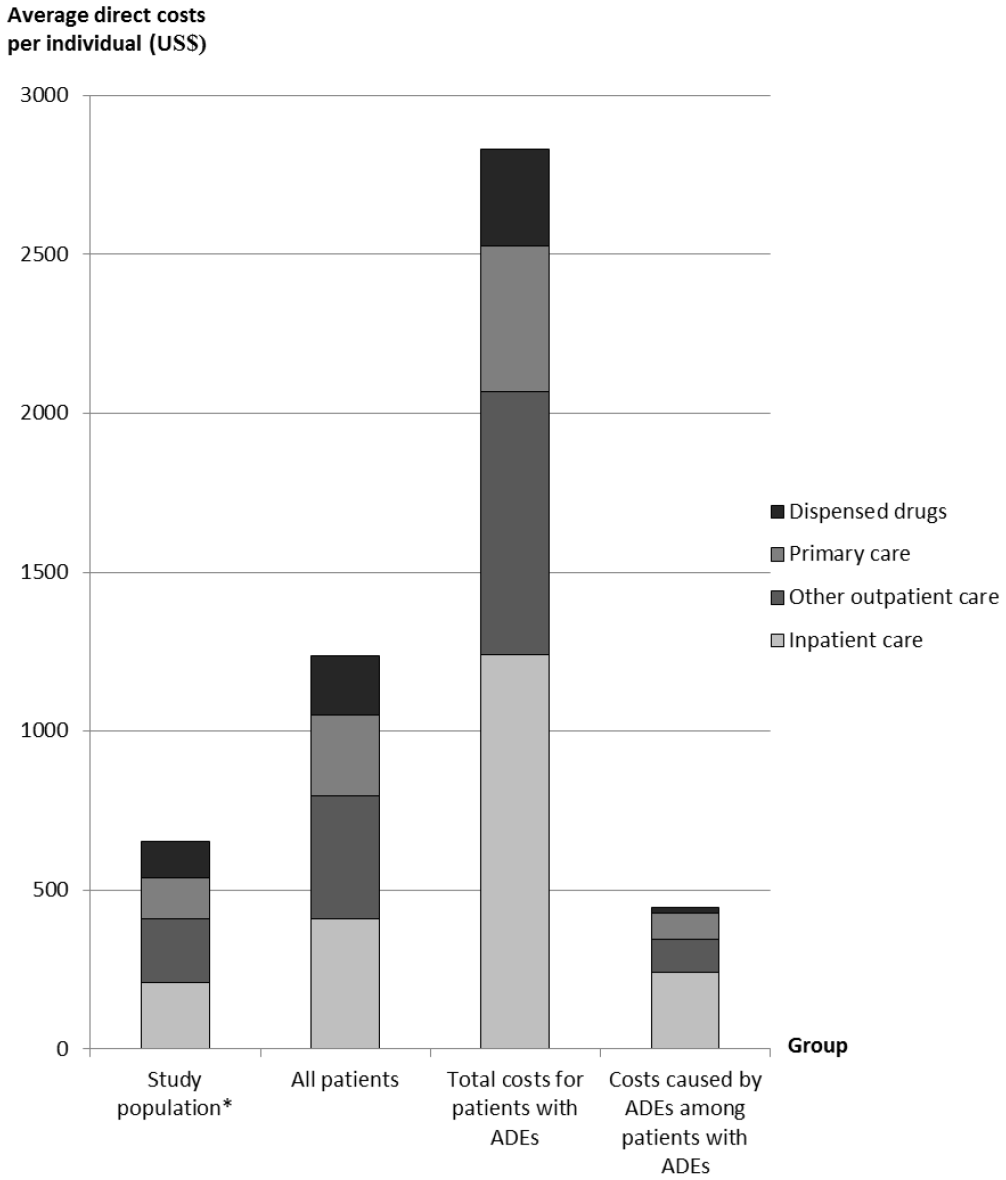
The direct costs for healthcare and drug use in Sweden. Average direct cost per individual during three months, by care level and resource type. * Summary measure for all individuals with and without ADEs in the study population (n = 4970).

Among the 596 patients with ADEs, the average direct costs caused by ADEs and the direct and indirect components of the societal COI did not differ by patient characteristics (table 5), apart from lower indirect costs in patients above the Swedish retirement age (≥65 years). The proportions of direct costs by care level are presented in figure 3 for the entire study population, for all patients with healthcare encounters, for all patients with ADEs, and for direct costs caused by ADEs among patients with ADEs. In total, ADEs caused 9.5% of all direct healthcare costs in the study population.

**Table 5:** Direct costs caused by adverse drug events (ADEs) and the direct and indirect costs of societal cost of illness (USD) per patient with ADEs (n = 596), by patient and ADE characteristics.

		Societal cost of illness for patients with ADEs	
Patient and ADE characteristics	Direct costs caused by ADEs, per patient with ADEs	Direct costs per patient with ADEs	Indirect costs per patients with ADEs
	USD mean (95% CI)	USD mean (95% CI)	USD mean (95% CI)
**Totalcosts (n = 596)**	**444.9 (264.4 to 625.3)**	**2830.1 (2260.7 to 3399.4)**	**3404.9 (2899.3 to 3910.4)**
**Age**			
18–34 years (n = 73)	617.2 (20.1 to 1214.4)	1976.2 (1167.3 to 2785.1)	2583.0 (1425.5 to 3740.6)
35–64 years (n = 267)	354.3 (104.0 to 604.5)	2495.9 (1728.9 to 3262.8)	6894.2 (5975.7 to 7812.7)
≥65 years (n = 256)	490.2 (204.9 to 775.5)	3422.2 (2388.4 to 4455.9)	0
**Sex**			
Men (n = 238)	299.9 (183.5 to 416.3)	3230.1 (2143.0 to 4317.2)	3489.8 (2663.4 to 4316.3)
Women (n = 358)	541.2 (250.7 to 831.8)	2564.2 (1947.6 to 3180.8)	3348.4 (2707.7 to 3989.1)
**ADE category** *			
Adverse drug reactions (n = 342)	534.1 (271.9 to 796.2)	3581.4 (2705.4 to 4457.3)	3645.5 (2964.1 to 4326.8)
Drug abuse and drug dependence, combined (n = 20)	1607.5 (−603.0 to 3818.1)	3498.1 (929.3 to 6066.9)	7623.6 (3940.6 to 11306.6)
Drug intoxication from overdose (n = 7)	546.3 (−191.7 to 1284.3)	3641.2 (−2130.1 to 9412.4)	2403.4 (−3387.1 to 8193.9)
Sub-therapeutic effect of drug therapy (n = 320)	592.6 (284.3 to 900.9)	2848.9 (2152.3 to 3545.4)	3382.6 (2693.6 to 4071.5)
Morbidity due to drug-related untreated indication (n = 47)	719.9 (53.6 to 1386.2)	2531.2 (1160.0 to 3902.3)	3178.5 (1318.6 to 5038.4)
**Preventabilityof ADEs**			
At least one preventable ADE (n = 278)	525.2 (263.8 to 786.7)	3016.0 (2263.1 to 3769.0)	3291.0 (2543.1 to 4039.0)
No preventable ADEs (n = 318)	374.6 (124.3 to 625.0)	2667.5 (1824.3 to 3510.8)	3504.4 (2815.1 to 4193.7)

* Each category includes all patients and estimated costs of patients with at least one ADE in the category, thus the figures will exceed the number of patients with ADEs and costs.

### Sensitivity analysis

For the 2224 healthcare encounters assigned a DRG in the register, our comparison showed that the average DRG weight was higher for encounters among those with ADEs compared to patients without ADEs (0.112, 0.086 to 0.138 vs. 0.068, 0.060 to 0.076) during the study period (p = 0.002). Among patients with ADE, the corresponding difference was not statistically significant between encounters dominantly caused by ADEs compared to other encounters (average DRG weight per encounter: 0.129, 0.094 to 0.163 vs. 0.097, 0.060 to 0.135, p = 0.223).

## Discussion

Our population-based study demonstrates that ADEs cause significant costs in healthcare, as the direct costs caused by ADEs during a three-month period were USD 445 per patient with ADEs. We found that 44% of the direct costs caused by ADEs occur outside inpatient care settings. The total societal COI per patient with ADEs, USD 6235 during the study period, was high compared to the COI of patients without ADEs. Indirect costs constituted half of the societal COI for patients with ADEs.

The main strengths of this study were the population-based random sample of adults in Östergötland, which is reasonably representative of all adults in Sweden.[7] Moreover, medical records from primary care, other outpatient care and inpatient care, as well as data from registers were used. Thus, we could examine ADEs and costs in detail from several aspects, including direct and indirect costs. The main limitation of the study was its retrospective design, as ADEs may have been overseen due to incomplete information in medical records. Moreover, sick-leave caused by ADEs could not be identified from the medical records, hindering estimation of indirect costs caused by ADEs. Another limitation was that the direct costs for diagnosing, treating and monitoring ADEs were limited to costs that could be identified in the administrative costs data and were therefore probably underestimated. For many of the identified interventions for diagnosing, treating and monitoring ADEs there were no intervention-specific costs available in the registers. As some of these encounters were associated with ADEs, our study underestimated the economic impact of ADEs and our results should therefore be viewed as minimum direct costs caused by ADEs in Sweden.

Our estimated cost per patient caused by ADEs was low compared to the costs identified in previous studies of ADEs in single-episode hospitalisations.[5] However, limited to patients with hospitalisations dominantly caused by ADEs (USD 6209), our results were comparable to published costs per ADE-related hospitalisation (EUR 3105  =  USD 3856,[15] in 2004),[17] and cost increases resulting from inpatient ADEs (USD 3420, in 2005–2006).[18] Moreover, our estimated proportion of emergency department visits dominantly caused by ADEs (18%) was comparable to previous estimates of 12%[19] and 28%.[20] However, we found that 16% of hospitalisations were dominantly caused by ADEs, which is higher than previously reported estimates (1% of admissions caused by ADEs,[17] and 11% of admissions associated with ADE[18]). The difference may result from relatively few hospital beds per capita in Sweden,[21] making patients more severely ill when hospitalised, and at a higher risk of complications (such as ADEs). Our estimated direct costs resulting from ADEs were high compared to a study of self-reported ADEs in Sweden.[6] This may be explained by survey respondents reporting fewer healthcare encounters than expected from healthcare use in the general population. As most previous studies have investigated costs using single care episodes and excluded outpatient care,[5] or lacked generalizability due to non-response,[6] our study with costs of all healthcare encounters in the general population provides more comprehensive information on the direct costs caused by ADEs than previous studies.

There are, to our knowledge, no prevalence- or incidence-based COI studies on ADEs, other than a survey study on the societal COI for survey respondents with self-reported ADEs.[6] Compared to the survey study, our estimated societal COI for patients with ADEs was relatively high (USD 6235 vs. USD PPP [purchasing power parity] 1500[6]), probably due to a shorter study period in the survey study and relatively few healthcare encounters among the survey respondents, as mentioned before. As in the survey study,[6] the higher societal COI for patients with ADEs compared to patients without ADEs was expected in our current study, considering the reported association between healthcare utilisation and ADEs.[4], [5] Similarly to the survey study,[6] the indirect costs in this study represented a large proportion (55% and 33%,[6] respectively) of the societal COI for patients with ADEs. Thus, our study adds to the literature that patients with ADEs have high societal COI, including high indirect costs. Moreover, the high indirect costs for patients with ADEs and the survey respondents' previously reported sick-leave and informal care caused by ADEs[6] suggest that part of the high indirect costs are caused by ADEs, although this could not be assessed from the medical records. The slight difference between the studies may be the result of both disproportionately healthy respondents to the survey (with e.g. relatively low healthcare use), and to the identification through medical records in the current study which excluded individuals with ADEs outside the healthcare system.

## Conclusions

In summary, our estimated direct healthcare costs caused by ADEs represented approximately 10% of the total direct healthcare costs in a random sample of adults in a Swedish county. Additional studies are therefore needed to identify interventions to prevent and manage ADEs. This includes research to further analyse the processes involved in interventions for diagnosing, treating and monitoring ADEs during healthcare encounters. Moreover, nearly half of the direct costs of ADEs in the society occurred in outpatient settings. The economic impact of ADEs will hence be underestimated in studies focusing on inpatient ADEs alone. In addition, there is a need for studying a broader variety of costs and outcomes resulting from ADEs, such as productivity loss, to ensure all relevant consequences are acknowledged. The high proportion of indirect costs in the societal COI for patients with ADEs suggests that the observed costs caused by ADEs would be even higher if indirect costs were included in this study.
